# Clinical and molecular analysis of smoothened inhibitors in Sonic Hedgehog medulloblastoma

**DOI:** 10.1093/noajnl/vdab097

**Published:** 2021-07-07

**Authors:** Victor Pereira, Jacob Torrejon, Dulanjalee Kariyawasam, Pablo Berlanga, Léa Guerrini-Rousseau, Olivier Ayrault, Pascale Varlet, Arnault Tauziède-Espariat, Stéphanie Puget, Stéphanie Bolle, Kevin Beccaria, Thomas Blauwblomme, Laurence Brugières, Jacques Grill, Birgit Geoerger, Christelle Dufour, Samuel Abbou

**Affiliations:** 1 Department of Pediatric Haematology and Oncology, Besançon University Hospital, Besançon, France; 2 Department of Pediatric and Adolescents Oncology, Gustave Roussy Cancer Institute, Paris Saclay University, Villejuif, France; 3 Curie University Institute, CNRS UMR 3347, INSERM U1021, Orsay, France; 4 Pediatric Endocrinology, Diabetology and Gynecology Department, Necker Enfant-Malades University Hospital, AP-HP, Paris, France; 5 Imagine Institute, Inserm U1163, Paris, France; 6 Cochin Institute, Inserm U1016, Paris, France; 7 INSERM, Molecular Predictors and New Targets in Oncology, Paris-Saclay University, Villejuif, France; 8 Department of Neuropathology, Saint-anne Hospital, Paris, France; 9 Department of Pediatric Neurosurgery, Necker Enfants-Malades University Hospital, AP-HP, Paris, France; 10 Department of Radiation Oncology, Gustave Roussy Cancer Institute, Paris Saclay University, Villejuif, France

**Keywords:** growth plate fusion, medulloblastoma, smoothened inhibitor, Sonic Hedgehog

## Abstract

**Background:**

Smoothened inhibitors (SMOi) have shown activity in Sonic Hedgehog (SHH) medulloblastoma, however this therapeutic class was not developed in children due to severe effects reported on growth. We hereby report long-term follow-up of young patients treated with SMOi for recurrent medulloblastoma.

**Methods:**

Clinical data on response and toxicity from patients treated with vismodegib or sonidegib from 2011 to 2019 for a SHH medulloblastoma were retrospectively reviewed. Methylation analysis and whole exome sequencing were performed whenever possible.

**Results:**

All patients with a somatic *PTCH1* mutation responded to SMOi (6/8), including 2 prolonged complete responses. One patient was free of disease 8.2 years after treatment. SMOi was challenged again for 3 patients. Two of them had a response, one with SMOi alone, the other one in combination with temozolomide despite previous progression under monotherapy. SMO resistance mutations were found in patients from biopsy at relapse. Combination with temozolomide or surgery plus radiotherapy was associated with very long disease control in 2 patients. The most severe adverse events were myalgia and growth plate fusion with metaphyseal sclerosis. Normal growth velocity was recovered for 1 patient although her final height was below estimated target height.

**Conclusions:**

Targeting SMO in mutated *PTCH1* is an interesting strategy for long-term responses. Combination of SMOi with chemotherapy or surgery and local radiotherapy is an appealing strategy to prevent early resistance and diminish SMOi exposure, especially in young patients. Inhibition of SHH pathway causes growth and development impairment but partial recovery of the growth velocity is possible.

**Key Points**

- Targeting SMO in mutated *PTCH1* medulloblastoma with prolonged responses.
- Combination of SMOi with chemotherapy or local treatment can overcomes resistance.
- Long-term outcome of patients treated with SMO inhibitors: partial growth recovery is possible after growth impairment.

**Importance of the Study**

We hereby report 8 young patients who received smoothened inhibitors (SMOi) for Sonic Hedgehog (SHH) medulloblastoma relapses, with long-term follow-up, and molecular analysis including methylome clustering and whole exome sequencing. This is the first study that report long-term outcomes of patients treated with SMOi. We report resistance mechanism and that combination of SMOi with local treatment or chemotherapy can lead to a prolonged response or overcome acquired resistance. The use of SMOi in pediatric population has been discontinued after the description of growth impairment. This study brings for the first time, new informations about the use of SMOi in pediatric population since the phase I study. We think that it will help physicians to make decision in relapsed SHH medulloblastoma, for which very few therapeutic options are available.

The group of medulloblastoma characterized by SHH pathway activation mostly affects patients of less than 5 years old or more than 16 years old and account for 30% of medulloblastomas, mostly with a desmoplastic/nodular histological phenotype.^1,2^ Hedgehog pathway activation can be driven by different mutations causing among others, a loss of function of PTCH1 (43% patients), a gain of function of SMO (9%), or a loss of function of SUFU (10%).^1–3^ Alterations in genes within the SHH pathway are frequent in infant and young children.^4^ Constitutive activation of SHH pathway, known as Gorlin syndrome, is associated with malignancies, such as basal cell carcinomas (BCCs) and medulloblastomas, or benign tumors.^5–8^ SHH-activated desmoplastic medulloblastoma or medulloblastoma with extensive nodularity (DMB/MBEN) of early childhood have a favorable prognosis when treated with radiation-sparing approaches.^9,10^ However, survival rates were excellent only for patients with SHH-activated DMB/MBEN if treatment included either intraventricular methotrexate or high-dose chemotherapy.^11–13^ Omission of both led high rates of relapses.

Smoothened inhibitors (SMOi) have shown efficacy in BCC leading to FDA and EMA approval of vismodegib or sonidegib.^14,15^ Activity in relapsed SHH medulloblastoma was reported but the best way to implement SMOi in the treatment of SHH medulloblastoma remains to be defined.^16–18^ Long-term toxicity of current treatment such as radiotherapy, especially in young children, is of great concern and has justified the study of new-targeted therapies. The SHH pathway is known to be of major importance in growth plate development and accelerated growth plate maturations were reported in preclinical studies and clinical trials, questionning the balance between tumor control and long-term sequelae.^18,19^

In this study, we report very prolonged responses and long-term follow-up of medulloblastoma patients treated by SMOi. We describe patients’ molecular alteration and resistance mechanism. These data are crucial for elaborating a solid treatment strategy that integrates tumor evolution, molecular biology, and long-term outcome for those rare patients.

## Methods

### Population

We retrospectively reviewed clinical, histological, and radiological files of the 8 children treated in Gustave Roussy—France, with a SMOi, from March 2011 to December 2019 for a recurrent medulloblastoma. SHH pathway activation was established by 2 methods including immunohistochemistry, CHG array, methylome profiling, or mutation in the pathway genes.^20^ Parents or guardians gave their written informed consent for retrospective analysis according to the institutional review board of Gustave Roussy. For patients enrolled in ongoing precision medicine protocols, parents or legal guardians gave their written informed consent. Our study follows the principles outlined in the “World Medical Association Declaration of Helsinki: Research involving human subjects.” Follow-up was updated on 01/01/2020.

### Treatment

Patients received orally either vismodegib or sonidegib at the recommended phase 2 dose. Safety and tumor responses were previously partially reported.^18^ Two patients received the treatment within a clinical trial (NCT01125800); the other 6 received their treatment as a compassionate use or off label. SMOi was discontinued for adverse event, progressive disease (PD), or patient’s decision. Other treatment such as temozolomide, arsenic trioxide, ribociclib, or other conventional chemotherapy was used off label after validation in a clinical tumor board.

### Response Evaluation

Brain and spinal magnetic resonance imaging were used and radiologic response was assessed according to the International Society of Pediatric Oncology criteria.^21^

### Toxicity

Adverse events were reported according to Common Terminology Criteria for Adverse Effects 4.0. Greulich and Pyle bone age (G&P) and knee X-rays were analyzed when available.

### Molecular Analysis

CGH array was performed for 2 patients as routine analysis using SurePrint G3 Human CGH Microarray Kit G4449A (Agilent). Whole exome sequencing were performed within MOSCATO or MAPPYACTS trial (respectively NCT01566019 and NCT02613962) for 5 patients.^22^ Whole-genome DNA methylation data of 4 samples presented in this study were generated from flash-frozen tumors using either Illumina Infinium Methylation EPIC or HumanMethylation450 BeadChip arrays (850k and 450k arrays, respectively) in accordance with manufacturer’s instructions at the DKFZ Genomics and Proteomics Core Facility. MB subgroup (WNT, SHH, G3, and G4) and SHH subtype affiliation (SHH INF and SHH CHL AD) predictions were first obtained from a DNA methylation-based classification web-platform for central nervous system tumors (www.molecularneuropathology.org, version 11b4).^23^

Beta values matrix. Raw data files (.idat) generated by the Illumina iScan array scanner were processed in the R statistical environment (v3.6.2) using the minfi (v1.32.0).^24^ The data were normalized using the preprocess Illumina method (background subtraction and control normalization) as part of the minfi package. For the downstream analysis (PCA, UMAP, and HC), we preprocessed additional set of 100 DNA methylation samples (450k arrays) from 2 reported works.^4,25^ The previously published data are available in the GEO under the accession numbers GSE85218 and GSE104728. Unsupervised clustering was conducted according to the main 4 MB subgroups (WNT, SHH, G3, and G4) previously defined by DKFZ classifier. For SHH subtype clustering (SHH INF vs SHH CHL AD), we have selected the top 4000 most variably methylated probes.

### Methylation Array Copy Number Analysis

Copy number profil was infered from methylation arrays by identification of broad and focal events. Copy number segmentation was performed from genome wide methylation arrays using the consume package (v1.20).^26^ Identification of broad and focal copy number events was performed from segmented copy number derived following the same criteria previously described.^27^

## Results

### Population

Between March 2011 and December 2019, 8 children, 4 females, and 4 males, received a SMOi for a medulloblastoma recurrence or progression with SHH pathway activation at Gustave Roussy. Clinical characteristic at diagnosis is reported in Table 1. At diagnosis, 6 had localized disease, and 2 had metastatic disease with leptomeningeal dissemination. Histological subtypes were DMB/MBEN for 6 children, and 1 case of none other specified medulloblastoma. One patient did not have any available tumor material and the diagnosis of SHH medulloblastoma was made based on the referring hospital report. Median follow-up was of 26.5 months (range 2–98).

**Table 1.** Smoothened Inhibitor Treatment

Treatment Before SMOi							SMOi Treatment Period					
#	Age at Diagnosis (y)	First-Line Treatment	Other Medical Treatment	Surgery	RT	Lines Before SMOi	Initial SHH Pathway Alteration	Age at SMOi	First SMOi Duration (m)	Clinical Improvement	Best Response	Discontinuation Cause
1	8	VP16/Carboplatine	TOTEM Regorafenib RAPIRI	Complete at diagnosis and relapse	Relapse	5	SHH activated on CGH TP53 C176W	12.5	1.2	None	PD	PD
2	5	M-SFOP 2007	TOTEM RAPIRI	Complete at diagnosis and relapse	Diagnosis and relapse	4	SSH activated on CGH TP53 del	7.9	3.4	Morphine and steroids decrease	PD	PD
3	4	HITSKK	VP16/Carboplatine	Complete	None	2	PTCH1 p.Q371HfsX65 germline	6.3	4.7	Neurological improvement	PR	PD
4	2	HITSKK	None	Partial at diagnosis	None	1	PTCH1 p.R135X	3.3	9.3	No symptom	PR	PD
5	18	VP16/Carboplatine	Temodal	Complete at diagnosis and relapse	Diagnosis and relapse	3	No data	25.5	5.6	No improvement	PR	AE
6	13	PNET HR+5	None	Partial at diagnosis	Diagnosis	1	PTCH1 p.L517PL	16.7	8.8	Headaches and asthenia improvement	PR	AE
7	2	VP16/Carboplatine	VP16/Carboplatine Busulfan/Thiotepa	Complete at diagnosis and relapse	Relapse	4	PTCH1 p.V403W	11.5	8.3	No symptom	CR	AE
8	3	HITSKK	None	Complete at diagnosis	None	1	PTCH1 p.E263K germline	4.9	9.4	No symptom	CR	AE

AE, adverse effects; CR, complete remission; CT, chemotherapy; PD, progressive disease; PR, partial remission; RT, radiotherapy; SHH, Sonic Hedgehog; SMOi, smoothened inhibitor.

### Front-Line and Relapse Treatment Prior to SMOi

Details of patients’ first-line and relapse treatment are provided in Table 1. Depending on their disease status at diagnosis, they were treated according to HIT SKK 92 protocol,^9^ MSFOP 2007,^28^ PNET HR+5,^29^ and HIT-SIOP PNET 4.^30^

### SHH Pathway Alteration

**Figure 1.**
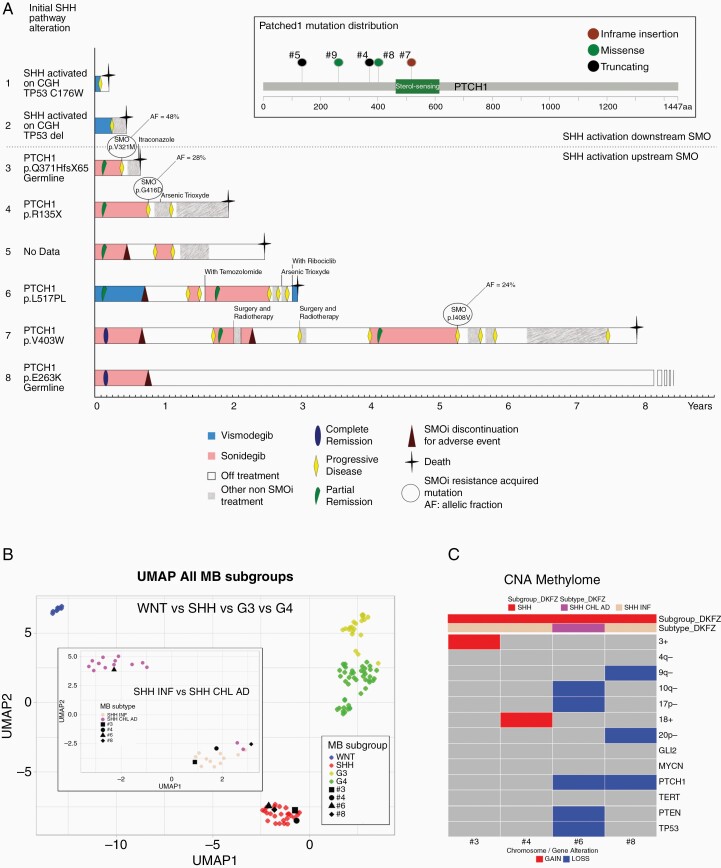
Patients’ treatment and molecular alteration. Swimmer plot representing patients’ outcome starting at the beginning of the first SMOi treatment (a). Patients are segregated by SHH activation downstream SMO, above the dotted line, or SHH activation upstream SMO, below the dotted line. The first column described initial SHH pathway molecular alteration based on CGH, methylome analysis, and/or sequencing. Event, including responses, progressive disease, discontinuation, or death are represented on the patient line as well as other relevant treatment targeting SHH pathway or resistance. For patients who underwent a new biopsy at progression, SMO mutation are reported with their allelic fraction in the tumor biopsy. Unsupervised clustering of methylation pattern according to medulloblastoma subgroups using UMAP showed that the 4 patients were clustering with the previously published SHH medulloblastoma (b). SHH subgroups affiliations between infant (INF) or child and adolescent (CHL AD) of patients tumors were consistent with age at diagnosis. Patient #6 was 13 years old while the remaining 3 patients were of 4 years old or below at diagnosis (c). copy number profils infered from methylation array showing recurrent copy number alteration in SHH medulloblastoma. SHH, Sonic Hedgehog; SMOi, smoothened inhibitor.

Five patients had a *PTCH1* alteration and among them, 2 were associated with the same germline alteration (Figure 1a). Mutated pathogenic PTCH1 protein p.R135X and p.V403W were previously described.^31^ Patient #3 had a frameshift germline mutation resulting in a nonfunctional PTCH1 protein, which is concordant with the clinical observation of a Gorlin syndrome. Patient #8 had a somatic and germline missense mutation c.787G>A. Despite the lack of evidence of a loss of function of PTCH1 protein, this mutation was reported once to be associated with a Gorlin syndrome (dbSNP: rs1588610385). Patient #6 was diagnosed with a somatic mutation p.L517PL. Interestingly, those 2 last patients harboring a *PTCH1* mutation of unknown significance, had a loss of the wild-type allele, which is in line with pathogenic alteration. Two patients without PTCH1 mutation carried a TP53 alteration.

Methylome analysis was performed for 4 patients. For all of them, unsupervised clustering of methylation pattern were consistent with SHH subgroup, 3 with SHH infant (#3, #4, and #8) and 1 with adolescent (#6) (Figure 1b and c).

### Response to the Treatment With the SMOi

**Figure 2.**
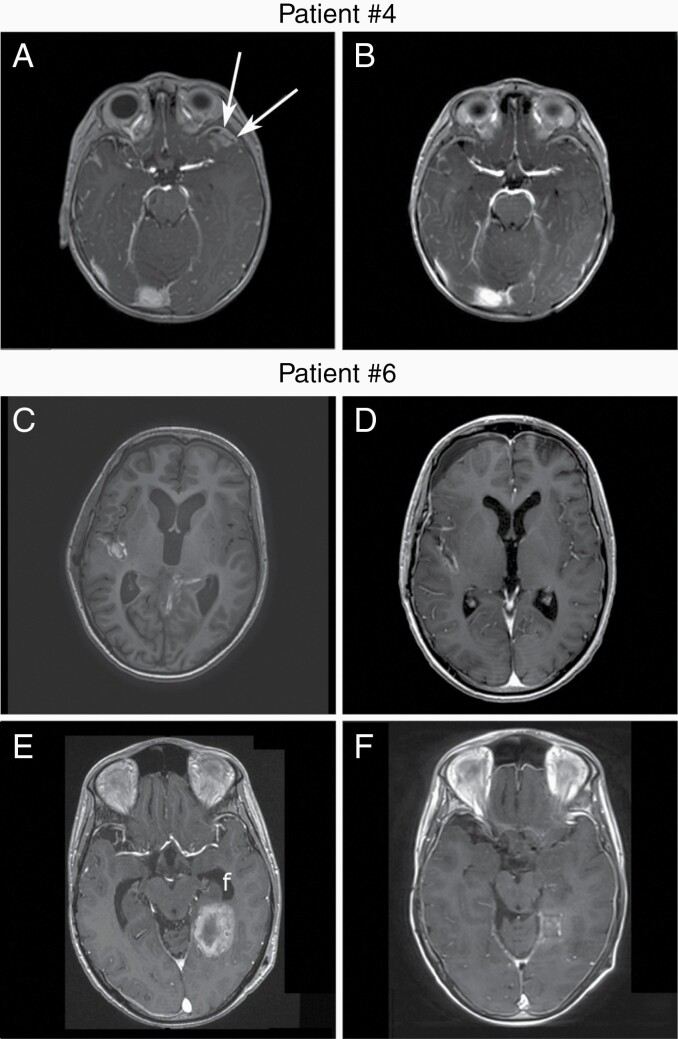
SMOi response evaluation by MRI T1 with contrast enhancement. Patient #4 baseline MRI before SMOi initiation. Contrast enhancement of a relapse in the temporal lobe (a) showing a very good partial response after 8 weeks of sonidegib (b). Patient #6 baseline MRI before SMOi initiation showing a metastatic relapse in the Silvius fissure (c). Partial response was achieved after 2 months of vismodegib (d). A Dramatic relapse occurred while treated by sonidegib, with new metastasis in the temporal lobe (e). Partial response was achieved gain 4 months later with a combination of sonidegib and temozolomide (f). Importantly, the patient had previously received temozolomide. MRI, magnetic resonance imaging; SMOi, smoothened inhibitor.

The SMOi vismodegib or sonidegib were used for 3 and 5 patients, respectively (Figure 1a). Median age at SMOi initiation was 11.1 years (range 3.3–25.5). Treatment duration was of 1.2–9.4 months, with a median duration of 7 months. All 5 patients with a known *PTCH1* mutation responded to a SMOi. Patient #5 responded while no information of *PTCH1* status is available. There were 6 (75%) objective responses, 4 partial remission (PR), and 2 complete remission (CR) (Figure 2, Supplementary Figure 1 and Table 1).

Disease progression was the first cause of treatment discontinuation with a median duration of 4 months (range 1.2–9.3) in 4 cases (50%). Discontinuation for intolerable adverse event happened in 4 (44%) cases with a median of 8.6 months (range 5.7–9.6).

Overall, 8 patients died with a median of follow-up of 23.5 months (1.1–95.1) after SMOi. One patient is still alive and free of disease after a CR under SMOi and no other treatment, with a follow-up of 8.2 years after SMOi initiation (patient #8).^18^

### SMOi Resistance and Rechallenge

Among the patients who had an initial response to SMOi, 5 experienced a PD while on treatment (Table 1 and Figure 1a). For 3 of them, a biopsy at progression was performed for a whole exome sequencing, allowing the identification of activating SMO mutations. All were already reported in patient with BCC treated with SMOi.^32–34^ Interestingly, patient #4 and #7 SMO mutations were a subclonal event.

Three patients had new attempts of SMOi treatment (#5, #6, and #7). The 2 patients for whom a combination of SMOi with other treatment, surgery, radiotherapy, or chemotherapy, had prolonged responses and several rechallenge of SMOi. Patient #6 had a PR with vismodegib during 8.8 months (Figure 2c and d) and after discontinuation for myalgia, a SMOi was rechallenged with sonidegib, with an initial and short clinical improvement quickly followed by a dramatic progression (Figure 2e). A combination of vismodegib and temozolomide was given with a very good PR, despite the fact that the patient already received temozolomide previously (Table 2 and Figure 2f). This response lasted for 11.3 months before a new PD. Among other conventional therapies, the GLI1 inhibitor, arsenic trioxide which inhibit SHH pathway downstream of SMO, was introduced without success. Finally, vismodegib was rechallenge in association with ribociclib without any response.

**Table 2.** Combinatorial Therapies

#	SMOi	Regimen	Combined Treatment	Regimen/Schedule	Local Treatment Modality	Treatment Duration (m)	Discontinuation Cause
6	Sonidegib	Continuous	Temozolomide	5 days/4 weeks	—	29.6	PD
6	Vismodegib	Continuous	Ribociclib	continuous	—	3.3	Death
7	Sonidegib	Continuous	Surgery	After PR	Complete resection	25.2	End of treatment
7	Sonidegib	Continuous	Radiotherapy	After PR	50 Gy on posterior fossa	25.2	End of treatment

PD, progressive disease; PR, partial response; SMOi, smoothened inhibitor.

Patient #7 had several long periods of disease control throughout 5 years after SMOi initiation. After a prolonged CR to sonidegib of 8.3 months, the treatment was discontinued for grade III growth plate maturation. A local relapse occurred a year later and was treated again with sonidegib resulting in a new PR. This treatment was consolidated by surgery and radiotherapy (Table 2). A new relapse occurred 1 year after discontinuation for myalgia, and was treated local control, allowing a period free of treatment of about a year before a new recurrence. Sonidegib was introduced for the third time with a good PR for 16 months before an activating SMO resistance mutation occurred.

### Toxicity

The majority (86.7%) of adverse events were grade I/II (Supplementary Table 1). Myalgia and cramp were commonly involved in treatment interruption.

**Figure 3.**
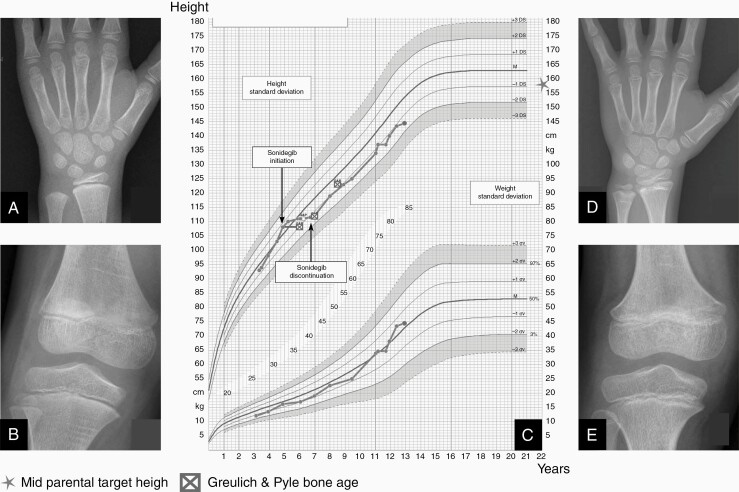
Patient #8 growth outcome. Patient #8’s Greulich and Pyle bone age (A) and knee X-rays (B) showing central epiphyseal fusion between sonidegib treatment baseline and 1 year after (D and E). Flattening of growth chart (C) from +0.5 SD to −1 SD during sonidegib treatment. After treatment withdrawal, growth velocity improved without recovering a normal growth.

**Figure 4.**
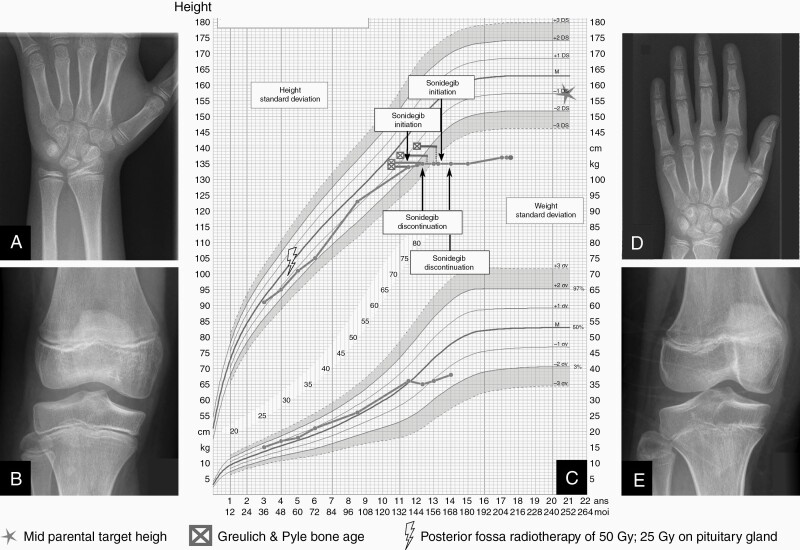
Patient #7 growth outcome. Patient #7’s Greulich and Pyle bone age (A) and knee X-rays (B) showing growth plate fusion between sonidegib treatment baseline and 1 year after with metaphyseal sclerosis (D and E). Flattening of growth chart (C) at sonidegib initiation with poor growth velocity at discontinuation. Her final height was 3 standard deviation below mid parent target height. To note, this patient had an ovarian dysfunction as a consequence of a high-dose chemotherapy that could impact her puberty.

The most severe adverse event was growth deceleration with growth plate fusion in all evaluable patients. Among the 4 patients who had not reached their adult height, all had grade III growth deceleration with complete growth arrest while on treatment. These growth arrests happened early after SMO initiation and was not secondary to premature bone maturation according the G&P assessments, as shown for patients #7 and #8 (Figures 3 and 4). On the contrary, expected growth plate ossification and phalanx epiphysis elongation slowed down during treatment initiation while patients experienced central epiphyseal fusion instead (Figures 3d, e and 4d, e). Patient #8 was 4 years and 9 months old at treatment initiation, P1 S1 on the Tanner scale and experienced a growth arrest resulting in a shift of growth channel from the +0.5 SD to −1 SD (Figure 3c). After SMOi discontinuation, she recovered a normal growth velocity on −1 SD curve. Seven years later, she had an impaired pubertal growth peak (only 7.5 cm) during pubertal development with a metaphyseal sclerosis. Moreover, she had premature apical tooth closure resulting in short roots and a severe mandibular prognathism that was detected 5 years after discontinuation, but the later could be related to the Gorlin syndrome.

Patient #7 was 11 years and 5 months with a height at −2 standard deviation (SD) at SMOi initiation. She was at P2 S1 on the Tanner scale. Her previous treatment included high-dose chemotherapy and radiotherapy with 25 Gy on the pituitary gland, resulting in ovarian dysfunction and growth hormone deficiency. She had a complete growth arrest on SMOi treatment leading to a growth shift from −2 SD to −4 SD and complete central growth plate fusion was seen on G&P and knee X-rays also showed metaphyseal sclerosis (Figure 4). She had no growth during pubertal development. The 2 other patients with a grade III growth retardation resulting in a growth arrest did not live long enough to evaluate long-term outcome.

## Discussion

This study is an integrated analysis of clinical and molecular biology data from 8 patients treated for a recurrent SHH medulloblastoma by a SMOi with a long-term follow-up. All the 6 patients with a *PTCH1* mutation had an objective tumor response and some of those responses were prolonged, especially when associated with other treatment. We reported SMO activating mutations at progression under SMOi. This treatment induces as expected long-term growth retardation and tooth development impairment.

Medulloblastoma treatment strategies try to avoid radiotherapy in young children.^11^ Nevertheless, relapse treatments, and above all radiotherapy after intraventricular methotrexate, have a dramatic negative impact on neurocognitive development and can be a cause of late mortality for long-term survivors.^11,29,35^ It is then crucial to limit the burden of treatment and to implement new treatment to improve outcome after a relapse that is often fatal.

Activation of SHH pathway is an appealing target since inhibitors of SMO were developed and approved for BCC.^7,14,15,36^ Here, we report 75% of pooled objective response rate and 100% in patients with somatic or germline *PTCH1* mutation. Response to SMOi was prolonged and disease control was for half of them over a year. Responses to SMOi in medulloblastoma were previously reported and as expected, only patients with SHH pathway activation upstream SMO had on objective response.^17,18,37–40^ This highlights the importance of assessing SHH pathway alteration prior to SMOi treatment.

Despite very good or complete responses, resistance to treatment oftenly occurred after at least 5 months of treatment. We found activating mutations of SMO in all the 3 patients with available material for sequencing. Those mutations were subclonal for 2 of them. Resistance mechanism involving SMO mutation are known in BCC, were described in medulloblastoma preclinical studies and only once in an extra-neural SHH medulloblastoma.^16,33,41,42^ However, a SMO p.G416D mutation—the same as patient #4—was described, concomitant with a PTCH1 loss of function, in a different case of an adult with SHH medulloblastoma without any previous SMOi exposure.^32^ One hypothesis to explain why that these 2 mutations can coexist, is that SMO activating mutation is an early subclonal event in PTCH1 mutated medulloblastoma, and that SMOi treatment selects a resistant clone. A recent single-cell transcriptome analysis on SHH medulloblastoma showed that cell diversity confers resistance to targeted therapy.^43^ Moreover, Petrirena et al. reported a patient with a PTCH1 mutated medulloblastoma who developed under vismodegib 2 different SMO mutations in 2 different metastatic sites at different allelic fraction.^42^

Combination therapy could be a way to prevent the selection of 1 or several resistant clones. Two patients of this serie had a prolonged response with a combination of a SMOi with either, a local control with surgery and radiotherapy, or temozolomide after a PD under single drug SMOi. Other drugs were described to be able to inhibit the growth of medulloblastoma cells in vitro as itraconazole or arsenic trioxide.^44^ A phase II randomized clinical trial combining temozolomide with vismodegib against temozolomide alone was conducted in adults patients with SHH medulloblastoma and did not report any improvement of 6-m PFS with the combination.^45^ Patients were not distributed in the different arms based on the molecular alteration. Only 11 patients with a proven PTCH1 mutation were randomized between arm A (combination of vismodegib and temozolomide, *n* = 4), arm B (temozolomide alone, *n* = 2), and arm C (vismodegib alone for patients previously treated by temozolomide, *n* = 5). Activation upstream SMO is important for SMOi response. Hence, with so few patients with a PTCH1 mutation, the study can not compare combination of SMOi with temozolomide or temozolomide alone in this specific population. Moreover, this trial only randomized adults patients which are of different medulloblastoma subgroup as pediatric and infant medulloblastoma. Further studies are required to explore combination to overcome resistance with inhibitors of SHH pathway or other pathway such as PI3k/Akt/mTor described to be involved in resistance.^46^ Nonstandard dosing schedules could be a way to prevent clone selection and were studied for various oral systemic anticancer therapies with limited evidence. However, some studies reported similar or improved responses. It would required preclinical proof of concept study and to be evaluated in a prospective trial.^47,48^

Time to progression with SMOi varied a lot between patients and one could ask whether the type of *PTCH1* mutation impacts response and resistance emergence. Among the 2 patients with single-nucleotide variation of uncertain significance in *PTCH1*, one is probably cured 8 years after the end of SMOi treatment, and the other one had several responses when rechallenged. This highlights the challenge to predict the relevance of an unknown mutation.

In this study, all evaluable patients had severe growth retardation and experienced epiphyseal fusion and sclerosis. We provide, for the first time, a long-term evaluation of children growth after SMOi treatment with an update on outcome for 2 patients reported in the sonidegib phase I study (patients #7 and #8).^18^ Interestingly, after SMOi withdrawal, 1 young patient (#8) was able to carry on her development and growth although with impaired final height despite a normal sexual development and puberty. The bone structure on X-ray suggests an impairment of bone formation with metaphyseal sclerosis. SHH pathway is crucial in fetal development, cellular proliferation and is also involved in chondrocytes differentiation, growth plate organization.^36,49^ Impact of SHH pathway alteration on bone structure has been also described in young mice and growth plate fusion were observed in patients in early phase trials of vismodegib and sonidegib.^18,19^ This is of major importance for patient management since growth hormone treatment is reported to cause osteonecrosis, and this risk can be higher in patients with bone disease.^50^ Finally, it is important to notice that early evaluation of mouth and tooth impairment was not able to predict late effect on tooth growth.

This work provides a strategic insight in how to manage patients with a relapse SHH medulloblastoma. Integration of molecular biology is important to anticipate resistance and to build combination therapy, with chemotherapy or other targeted inhibitors. SMOi must be used as much as possible on short period, as a bridge to local control and combination is a key to prevent early resistance. Inhibition of this pathway in young children causes growth and development impairment but it is possible to recover partially if the treatment is short enough in patients with a growth potential.

## Supplementary Material

Supplementary material is available at *Neuro-Oncology Advances* online.

Supplemental Figure 1: MRI at relapse and at remission obtention. MRI before (left panel) and after SMOi (right panel). Reduction of contrast enhancement after 2 (a), 8 (c), 3 (d), and 2 (e) months of treatment. FLAIR signal reduction (b) after 2 months of SMOi.

vdab097_suppl_Supplementary_Materials_S1

vdab097_suppl_Supplementary_Materials_S2

## Notes

**Authorship Statement.** Design the study, figures conception, data analysis, and writing: V. Pereira and S. Abbou. Figures and bioinformatic analysis, writing, and reviewing: J. Torrejon and O. Ayrault. Writing, figures, and reviewing (long-term toxicities): D. Kariyawasam. Histopathological review: P. Varlet and A. Tauziède-Espariat. Patients recruitment: L. Guerrini-Rousseau, L. Brugières, C. Dufour, J. Grill, S. Abbou, S. Bolle, S. Puget, K. Beccaria, T. Blauwblomme, P. Berlanga, and L. Brugières. Designed of study and paper reviewing: J. Grill, C. Dufour, and L. Guerrini-Rousseau.
