# Supplementary material for: Clinical and molecular analysis of smoothened inhibitors in Sonic Hedgehog medulloblastoma
Source: Neurooncol Adv. 2021 Jul 7;3(1):vdab097. doi: 10.1093/noajnl/vdab097 (PMC8367281; doi:10.1093/noajnl/vdab097)
Supplement: vdab097_suppl_Supplementary_Materials_S1 [file vdab097_suppl_supplementary_materials_s1.docx]

Grade I-II (%) Grade III – IV (%)

N = 9 patients

Alopecia

Hairs 3 (33)

Eyebrows 2 (22)

Drought

Skin 2 (22)

Mouth 2(22)

Muscles

Myalgia 4 (44) *

Elevated CPK 3 (33)

Cramps 3 (33) *

Digestive system

Abdominal pain 1 (11)

Vomiting 1 (11)

Neurology

Asthenia 3 (33)

Memory loss 1 (11)

Endocrinology

Growth retardation/Cartilage fusion 4 (44) **

Anorexia 1 (11)

Supplementary Table 1 Smoothened inhibitor treatment toxicity

** Non tolerable Grade II toxicity leading to SMOi discontinuation*

*** Grade III-IV toxicity leading to SMOi discontinuation*

*N: Number ; SMOi: Smoothened inhibitors*
