# Supplementary figures and images for: Clinical and molecular analysis of smoothened inhibitors in Sonic Hedgehog medulloblastoma
Source: Neurooncol Adv. 2021 Jul 7;3(1):vdab097. doi: 10.1093/noajnl/vdab097 (PMC8367281; doi:10.1093/noajnl/vdab097)

Patient

At relapse

PR/CR on therapy

a  
#3

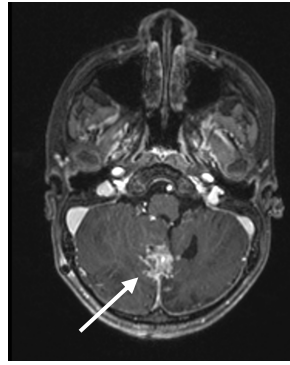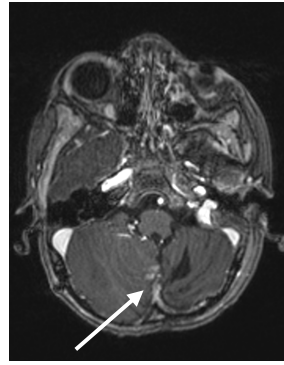

b  
#5

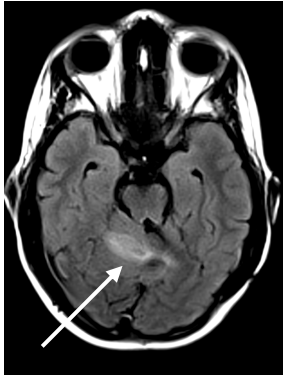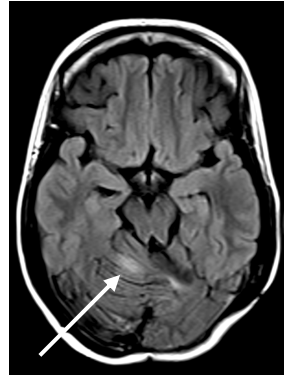

c  
#6

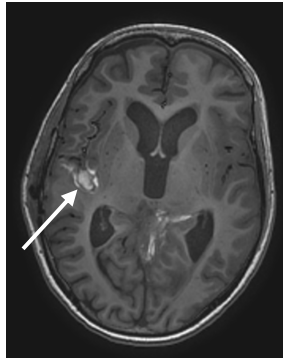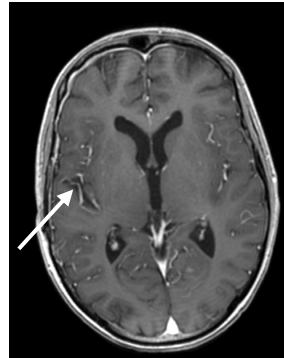

d  
#7

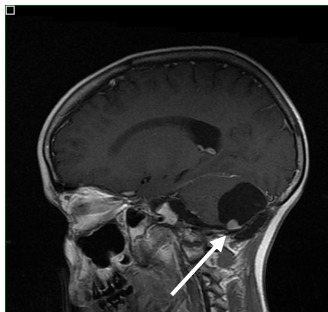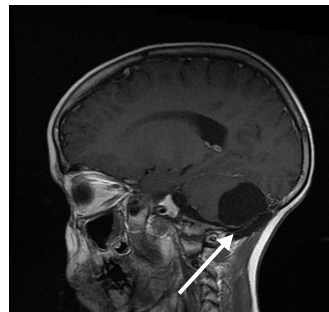

e  
#8

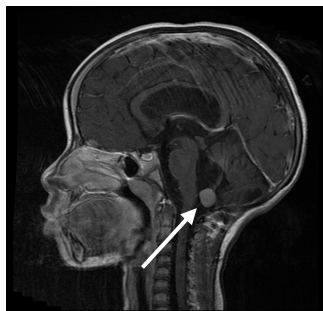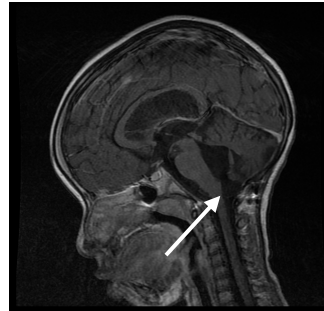

Supplement: vdab097_suppl_Supplementary_Materials_S2 [file vdab097_suppl_supplementary_materials_s2.pdf]
